# Supplementary material for: Dairy Wastewaters to Promote Mixotrophic Metabolism in Limnospira (Spirulina) platensis: Effect on Biomass Composition, Phycocyanin Content, and Fatty Acid Methyl Ester Profile
Source: Life (Basel). 2025 Jan 26;15(2):184. doi: 10.3390/life15020184 (PMC11856459; doi:10.3390/life15020184)
Supplement: Supplementary file 1 [file life-15-00184-s001.zip › life-3418823-supplementary.pdf]

Table S1: Experimental setup

	JM<br>mL	CW<br>mL	Inoculum<br>mL	Total volume<br>mL	CW in JM<br>%
CTRL	540	0	60	600	0
SW-1%	534	6	60	600	1
SW-2%	528	12	60	600	2
SW-4%	516	24	60	600	4
BMW-1%	554	6	40	600	1
BMW-2%	548	12	40	600	2
BMW-4%	536	24	40	600	4
DWW-1%	570	6	24	600	1
DWW-2%	564	12	24	600	2
DWW-4%	552	24	24	600	4

Note: JM = Jourdan medium, CW = cheese whey, CTRL = control JM, SW = scotta whey, BMW = buttermilk wastewater, DWW = final CW wastewater
